# Long-range connections damage in white matter hyperintensities affects information processing speed

**DOI:** 10.1093/braincomms/fcae042

**Published:** 2024-02-21

**Authors:** Tong Lu, Zan Wang, Yixin Zhu, Mengxue Wang, Chun-Qiang Lu, Shenghong Ju

**Affiliations:** Department of Radiology, Zhongda Hospital, School of Medicine, Southeast University, Nanjing 210009, China; Department of Neurology, Zhongda Hospital, School of Medicine, Southeast University, Nanjing 210009, China; Department of Neurology, Zhongda Hospital, School of Medicine, Southeast University, Nanjing 210009, China; Department of Neurology, Zhongda Hospital, School of Medicine, Southeast University, Nanjing 210009, China; Department of Radiology, Zhongda Hospital, School of Medicine, Southeast University, Nanjing 210009, China; Department of Radiology, Zhongda Hospital, School of Medicine, Southeast University, Nanjing 210009, China

**Keywords:** cerebral small vessel disease, white matter hyperintensities, cognitive impairment, connectome, functional connectivity

## Abstract

White matter hyperintensities, one of the major markers of cerebral small vessel disease, disrupt the integrity of neuronal networks and ultimately contribute to cognitive dysfunction. However, a deeper understanding of how white matter hyperintensities related to the connectivity patterns of brain hubs at the neural network level could provide valuable insights into the relationship between white matter hyperintensities and cognitive dysfunction. A total of 36 patients with moderate to severe white matter hyperintensities (Fazekas score ≥ 3) and 34 healthy controls underwent comprehensive neuropsychological assessments and resting-state functional MRI scans. The voxel-based graph-theory approach-functional connectivity strength was employed to systematically investigate the topological organization of the whole-brain networks. The white matter hyperintensities patients performed significantly worse than the healthy controls in episodic memory, executive function and information processing speed. Additionally, we found that white matter hyperintensities selectively affected highly connected hub regions, predominantly involving the medial and lateral prefrontal, precuneus, inferior parietal lobule, insula and thalamus. Intriguingly, this impairment was connectivity distance-dependent, with the most prominent disruptions observed in long-range connections (e.g. 100–150 mm). Finally, these disruptions of hub connectivity (e.g. the long-range functional connectivity strength in the left dorsolateral prefrontal cortex) positively correlated with the cognitive performance in white matter hyperintensities patients. Our findings emphasize that the disrupted hub connectivity patterns in white matter hyperintensities are dependent on connection distance, especially longer-distance connections, which in turn predispose white matter hyperintensities patients to worse cognitive function.

## Teaser

Lu *et al*. reported that the disrupted hub connectivity patterns in patients with white matter hyperintensities were connection-distance-dependent, with the most prominent disruptions appearing in the long-range connections (e.g. 100–150 mm).

Disrupted long-range connections were associated with information processing speed in patients with white matter hyperintensities.

## Graphical Abstract

**Graphical Abstract:**
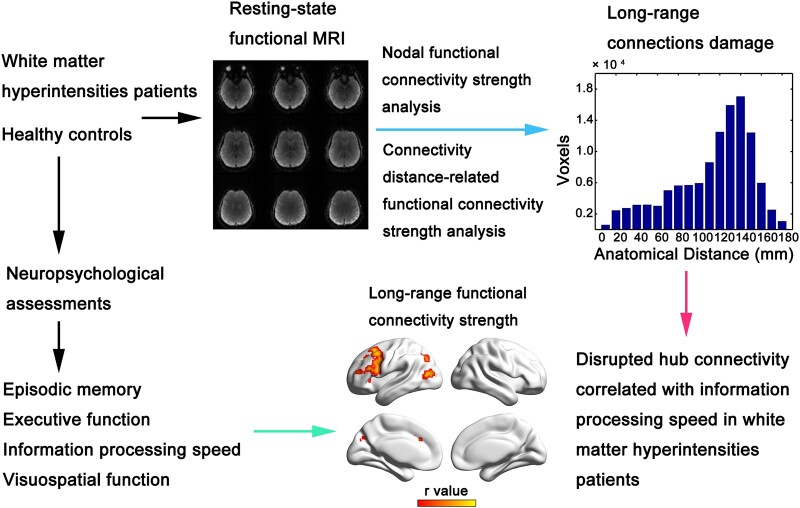

## Introduction

White matter hyperintensities (WMHs), a prominent characteristic of cerebral small vessel disease (CSVD), most commonly lie in periventricular and/or deep white matter (WM) regions on T_2_-weighted or fluid-attenuated inversion recovery (FLAIR) images.^1,2^ The prevalence of WMHs in older population is almost ubiquitous,^3,4^ and age is an important consistent risk factor for WMHs.^5^ More importantly, WMHs are linked to an increased risk of cognitive dysfunction^6,7^ and are indeed a leading cause of vascular cognitive impairment.^8^ In vascular cognitive impairment, memory functions are initially preserved, and the deficits predominantly affect executive function and information processing speed.^9^ While several studies have highlighted the association between WMHs and compromised cognitive function, the underlying neurobiological pathways mediating this relationship remain widely unknown.

The disconnection of distant brain regions leads to impaired network function and results in cognitive decline characteristic of vascular cognitive impairment. WMHs are usually identified as periventricular WM signal abnormalities, evoking network alterations of WM structural connections in patients with CSVD.^10,11^ CSVD has also been shown to impact multiple brain functional networks and reduce functional connectivity in previous functional MRI studies.^12^ Furthermore, in recent years, graph-theory analysis has gained growing popularity in the fields of neuroimaging and brain network analysis. It offers advanced tools for exploring the topological organization of brain networks, potentially providing valuable insights into the pathophysiology of CSVD.^13-18^ Graph-theory analyses have revealed that individuals suffering from CSVD often display increased path length and modularity, along with decreased network efficiency correlated with cognitive function (e.g. psychomotor speed) in the global topology of structural and functional whole-brain networks.^13,19^ These studies of brain connectomes underscore the importance of connectome-based research in enhancing our understanding of CSVD-related cognitive dysfunction at the whole-brain level.

For analysing the human brain connectome, network centrality analysis has recently emerged as a pivotal tool that captures the significance of individual brain regions based on their interconnectedness within the network.^20^ Brain connectome studies have demonstrated that human brain networks contain a small number of hubs with a significantly greater number of connections.^21^ These brain hubs exhibit elevated rates of cerebral blood flow, aerobic glycolysis and oxidative glucose metabolism and play a key role in facilitating rapid communication among various brain regions.^22,23^ As we know, metabolic costs in the brain rise in direct proportion to the total surface area of the neuronal membrane. Thus, these costs are contingent on axonal diameter and length, which are two pivotal factors in determining the surface area of the neuronal membrane, with longer-distance connections being more metabolically demanding to maintain.^24^ Additionally, because of the relatively lower blood flow, WM is usually more susceptible to damage when lesioned, compared to grey matter (GM);^25^ and long-range fibres are specifically susceptible to damage. Therefore, according to the pathological mechanism of WMHs, long-range brain hubs and connections may be more susceptible to WMHs-related impairment due to their heightened metabolic requirements. Additionally, since long-range fibres are supplied by a greater number of blood vessels, any blockages within these perfusion territories may lead to damage to axonal fibres.^26,27^ However, only a few studies have directly investigated whether WMHs are primarily linked to longer-distance disconnections or if WMHs-related disruptions in brain hubs are contingent on connection distance.

Hence, this present study aimed to comprehensively explore WMHs-related alterations in functional hubs within whole-brain networks using resting-state functional MRI (R-fMRI) and voxel-based graph-theory approaches. This voxel-wise approach eliminates the potential parcellation-dependent effects on the topological organization of brain networks.^20^ We aimed to determine: (i) whether individuals with WMHs exhibit disruptions in hub connectivity patterns within their whole-brain functional networks and whether these disruptions are connection-distance-dependent; and (ii) if so, whether these long-range disconnections mediate the relationship between WMHs and cognitive dysfunction, especially the information processing speed.

## Materials and methods

### Study participants

In this study, we initially recruited 70 elder, naturally right-handed Han Chinese individuals from a hospital, including 36 consecutive subjects with moderate to severe WMHs (defined as a sum of the deep WMHs Fazekas score and the periventricular WMHs Fazekas score ≥ 3 on FLAIR images) and 34 healthy controls (HCs) without WMHs (Fazekas score = 0). The exclusion criteria were as follows: (i) a history of ischaemic stroke with a diameter of >15 mm or cardiogenic cerebral infarction; (ii) a history of Alzheimer’s disease, Parkinson’s disease, epilepsy or head injury; (iii) a history of severe heart disease, liver disease, kidney failure or cancer; (iv) neuropsychological disorders; (v) WM lesions result from other causes (e.g. metabolism, immunity, toxicity, demyelination and infection); and (vi) MRI scan contraindications. This study was approved by the medical ethics committee of Zhongda Hospital (2019ZDSYLL189-P01). Written informed consent was obtained from all participants.

### Neuropsychological assessments

To evaluate cognitive performances, neuropsychological assessments were obtained in all participants within a week of their MRI scans. We assessed overall cognitive function for all participants using the mini-mental state exam and performed a concise cognitive test battery to measure multiple cognitive domains, e.g. episodic memory (auditory verbal learning test—20 min delayed recall, Rey–Osterrieth complex figure test—20 min delayed recall, logical memory test with a 20 min delayed recall), visuospatial function (clock drawing test, Rey–Osterrieth complex figure test), executive function (digit span test, trail making test B, verbal fluency test, Stroop C and semantic similarity test) and information processing speed (symbol digit modalities test, trail making test A, Stroop colour–word tests A and B). We further calculated composite *Z*-scores to represent the performance for each cognitive domain.^16^

### Multimodal MRI acquisition

All participants underwent multimodal MRI scans by a clinical 3.0 Tesla scanner (Siemens Healthcare, Germany) using a 12-channel standard head coil. A high-resolution 3D T_1_-weighted magnetization prepared rapid gradient echo (MPRAGE) sagittal sequence covering the whole brain was applied for anatomic reference using the following parameters: repetition time (TR) = 1900 ms, echo time (TE) = 2.48 ms, flip angle (FA) = 9°, matrix = 256 × 256, field of view (FOV) = 250 × 250 mm^2^, slice thickness = 1 mm, gap = 0 mm and slices = 176. R-fMRI images were collected for 8 min using a gradient-recalled echo-planar imaging sequence: TR/TE = 2000/25 ms, FA = 90°, matrix = 64 × 64, FOV = 240 × 240 mm^2^, slice thickness = 4.0 mm, gap = 0 mm and number of slices = 36. Additionally, axial T_2_-weighted FLAIR, diffusion-weighted images and susceptibility-weighted images were acquired to detect acute or subacute infarctions, perivascular spaces and cerebral microbleeds.

### Image preprocessing

The R-fMRI data preprocessing was carried out using the SPM8 (http://www.fil.ion.ucl.ac.uk/spm) and DPARSFA (http://www.restfmri.net/forum/dparsf) software. Briefly, the initial 10 functional volumes were discarded for participant acclimatization and scanner stabilization; and the remaining R-fMRI images were further corrected for timing differences and motion artefacts. Next, the individual structural images (T_1_-weighted MPRAGE images) were co-registered to the mean functional image using a linear transformation. The transformed structural images were subsequently segmented into GM, WM and cerebrospinal fluid using a unified segmentation algorithm. The motion-corrected functional volumes were further spatially normalized to the Montreal Neurological Institute space and resampled to 3 mm isotropic voxels using the normalization parameters estimated during unified segmentation. Then, band-pass filtering (0.01–0.1 Hz) was applied to the time series of each voxel, while linear trends were eliminated using a linear regression approach. Finally, multiple linear regression analysis was performed to remove the nuisance signals (six head motion parameters, mean global signal, cerebrospinal fluid signal and WM signal). Given that the removal of the global signal introduced a shift in the distribution of correlation coefficients (primarily the presence of negative correlations) and made the biological interpretation ambiguous,^28,29^ we restricted our investigations to positive correlations, consistent with prior studies.^20,30^

Notably, four WMHs patients were excluded due to excessive artefacts (i.e. translational motion more than 3 mm or rotational motion more than 3°) or incomplete acquisition, resulting in 32 WMHs patients and 34 HCs for further functional connectivity strength (FCS) analyses.

### Nodal FCS analysis

Nodal FCS analysis was performed using the GRETNA package (http://www.nitrc.org/projects/gretna/). First, for each subject, we created a whole-brain functional connectivity matrix by calculating the Pearson’s correlations between the time series of all possible pairs of brain voxels within a GM mask (*N*_voxels_ = 67 541), which was generated by setting a threshold of 0.2 on the mean GM probability map. Then, in order to quantify the functional integrity of the whole-brain network, voxel-based FCS analysis was further performed.^31^ For a given GM voxel, *i*, we computed its FCS using the following equation:


(1) $$\mathrm{FCS}(i)=\frac{1}{N_{\mathrm{voxels}}-1}\overset{N_{\mathrm{voxels}}}{\underset{\;j=1,j\neq i}{\sum }}\;z_{ij},r_{ij}>r_{0}$$

where *r_ij_* is the correlation coefficient between voxel *i* and voxel *j*, *r*_0_ is a threshold that is established to eliminate weak correlations possibly arising from signal noise (*r*_0_ = 0.2 in this study) and *r_ij_* is converted to *z_ij_* using Fisher’s *Z*-transformation when calculating FCS. Particularly, the FCS metric is referred to as the ‘degree centrality’ of a weighted network in graph theory. Following the above procedures, we obtained an individual FCS map for all participants.

### Connectivity distance-related FCS analyses

Given that age-related cerebral small vessel disease (featured partially by WMHs) may lead to a gradual decline particularly in the long-range WM fibre tract architecture, it becomes crucial to investigate whether WMHs-related alterations in FCS are influenced by the distance between brain regions. Of note, there is currently no established distance threshold for categorizing a specific functional connectivity as short- or long-range. In this study, we computed the Euclidean distance, *D_ij_*, as an approximate anatomical distance of functional connectivity between voxel *i* and voxel *j*. Subsequently, we segmented the whole-brain functional connectivity maps into 18 bins with Euclidean distances categorized into 10 mm intervals, spanning from 0–180 mm (the maximum distance between voxels in the GM mask). For a given voxel, *i*, the FCS at the *k*th bin (*k* = 1, 2, . . ., 18) was calculated as follows:


(2) $$\begin{array}{rl} \mathrm{FCS}(i,k) & =\frac{1}{N_{\mathrm{voxels}}-1}\underset{\;j\neq i,j\in D_{ik}}{\sum }\;z_{ij},r_{ij}>r_{0};\\ D_{ik} & =\{\;j|10\times (k-1)\leq D_{ik}<10\times k\} \end{array}$$

where *z_ij_* is the Fisher’s *Z*-transformed version of the correlation coefficient, *r_ij_*, between voxel *i* and voxel *j*, *N*_voxels_ is the number of voxels in the GM mask (*N*_voxels_ = 67 541), *r*_0_ in this study is 0.2 as mentioned above and *D_ij_* is the Euclidean distance between voxel *i* and voxel *j*. Consequently, for each subject, a new FCS map was generated for each distance bin.

## Statistical analyses

### Demographic data and cognitive performances

Statistical analyses of demographics were performed using independent two-sample *t*-test or chi-square tests for continuous variables and using chi-square tests for categorical variables. A one-way analysis of covariance (ANCOVA) was conducted to examine the between-group differences in neuropsychological *Z*-scores, with age and education as covariates. These analyses were implemented in SPSS 17.0 (SPSS, Inc., Chicago, IL).

### Between-group differences in FCS

A voxel-wise one-way ANCOVA was performed to investigate the between-group differences in full-range FCS, with age, sex and years of education as covariates. Notably, prior to statistical analysis, all individual FCS maps were spatially smoothed using a Gaussian kernel with a full width at half-maximum of 6 mm. The correction for multiple comparisons was conducted by Monte Carlo simulations and confined within the GM mask. The *α* level of 0.05 was obtained with a voxel-wise *P* < 0.05 and cluster size > 4590 mm^3^. The FCS metric enables the identification of brain regions displaying nodal functional connectivity alterations related to WMHs.

To further investigate between-group differences of FCS at each bin, ANCOVA analyses were conducted again on a voxel-wise basis, with age, sex and years of education as covariates, and multiple comparisons were corrected using Monte Carlo simulations. Importantly, since the FCS maps in different distance bins contained differing numbers of GM voxels, multiple comparison corrections were conducted within their respective masks.

### Association between FCS metrics and cognitive performances

To further investigate the associations between nodal FCS values in brain regions exhibiting significant between-group differences and neuropsychological composite *Z*-scores, we conducted voxel-by-voxel multiple linear regression analyses in the WMHs patients, adjusting for age, sex and years of education.

## Results

### Demographic data and cognitive performances

Demographic data and cognitive performances for all participants are summarized in Table 1. Although no significant difference was found in the years of education between the WMHs and HCs groups, the two groups were not matched for age and sex. Thus, age and sex effects were removed in all subsequent cognitive performance and FCS analyses.

**Table 1:** Demographic data and cognitive performance for all participants

	WMHs patients (*n* = 36)	Healthy controls (*n* = 34)	*P*/χ^2^-values
Demographics			
Age (years)	65.2 ± 10.1	71.0 ± 8.0	0.017^a^
Education (years)	11.0 ± 3.4	12.3 ± 3.5	0.141^a^
Sex (M/F)	17/19	7/27	0.019^b^
Cognitive performances			
MMSE	26.4 ± 3.7	28.3 ± 1.6	0.040^c^
Episodic memory	−0.32 ± 0.74	0.34 ± 0.63	0.009^c^
Executive function	−0.31 ± 0.70	0.33 ± 0.56	0.003^c^
Information processing speed	−0.41 ± 0.72	0.43 ± 0.70	0.003^c^
Visuospatial function	−0.07 ± 1.09	0.08 ± 0.56	0.635^c^

Data are presented as mean ± standard deviation. The cognitive performance in each domain is represented as composite *Z*-scores. WMHs, white matter hyperintensities; MMSE, mini-mental state examination; M/F, male/female.

^a^Independent-sample *t*-test.

^b^χ^2^-test.

^c^Analyses of covariance.

The WMHs group exhibited significantly worse than the HCs group in episodic memory (*P* = 0.009), executive function (*P* = 0.003) and information processing speed (*P* = 0.003).

### Between-group differences in whole-brain functional connectivity

**Figure 1:**
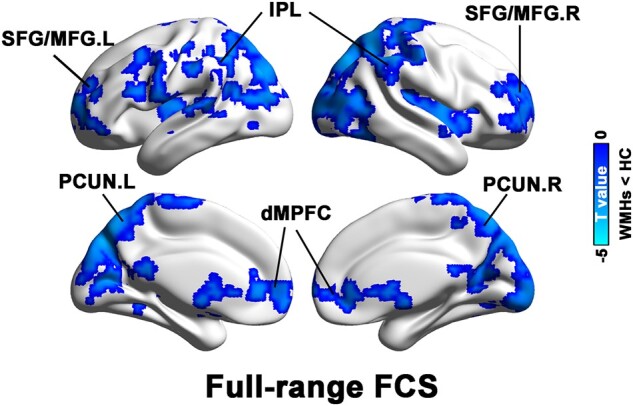
**Between-group differences in full-range FCS.** Compared with the HC subjects, the WMHs patients showed significantly decreased FCS mainly located in the bilateral superior/middle frontal gyrus (SFG/MFG), dorsal medial prefrontal cortex (dMPFC), precuneus (PCUN), inferior parietal lobule (IPL), insula, thalamus, calcarine fissure and surrounding cortex and superior/middle occipital gyrus. FCS, functional connectivity strength; HC, healthy control; WMHs, white matter hyperintensities.

Compared with HCs, the WMHs patients showed significantly decreased FCS mainly located in the bilateral superior/middle frontal gyrus, dorsal medial prefrontal cortex, precuneus (PCUN), inferior parietal lobule (IPL), insula, thalamus, calcarine fissure and surrounding cortex and superior/middle occipital gyrus (Fig. 1).

### Distance-dependent FCS patterns in WMHs patients

**Figure 2:**
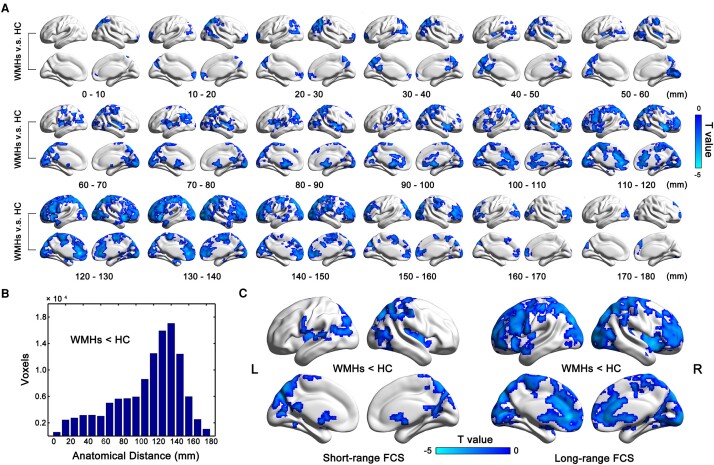
**Distance-dependent FCS patterns in WMHs.** (**A**) Between-group differences in different distance bins. The between-group FCS maps exhibited similar patterns in neighbouring distance bins, but were very different between very short and long distances. The reduced FCS values in WMHs were predominantly observed in the bilateral dorsolateral prefrontal cortex, medial prefrontal cortex, precuneus (PCUN), inferior parietal lobule (IPL), insula, thalamus and occipital lobe. Of note, the most prominent WMHs-related FCS decreases were observed within the 100–150 mm range, indicating that WMHs were primarily associated with longer-distance disconnections. (**B**) The number of voxels exhibiting significant group differences in FCS in different distance bins. (**C**) Difference maps between groups for short- and long-range FCS. We grouped the 18 FCS bins into two categories: 0–100 mm (short-range FCS) and 100–180 mm (long-range FCS). FCS, functional connectivity strength; WMHs, white matter hyperintensities.

Figure 2A illustrates between-group FCS maps for each examined connectivity distance. These maps exhibited similar patterns in adjacent distance bins but displayed significant differences between very short and long distances. The WMHs group exhibited reduced FCS values primarily localized in the bilateral dorsolateral prefrontal cortex, medial prefrontal cortex, PCUN, IPL, insula, thalamus and occipital lobe. It is noteworthy that the most pronounced FCS decreases associated with WMHs were observed in the 100–150 mm range (Fig. 2B), indicating a predominant association of WMHs with longer-distance disconnections.

### Association between the FCS patterns and cognitive performances in WMHs patients

**Figure 3:**
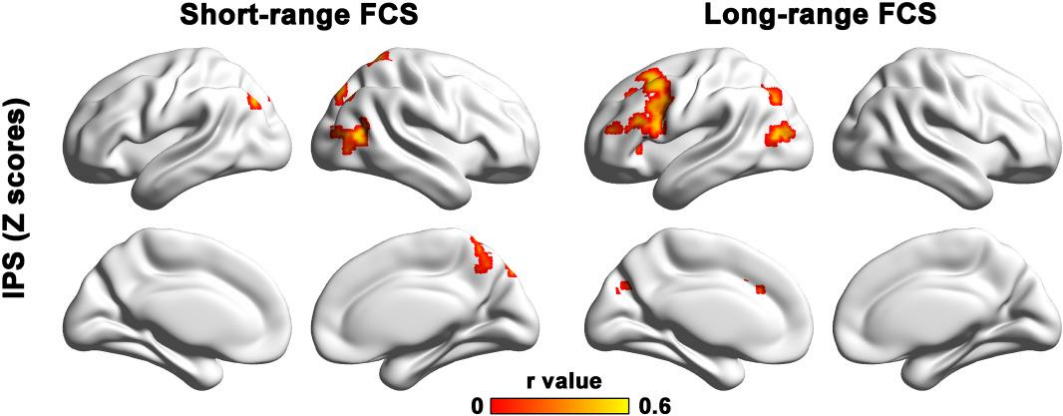
**Relationships between the cognitive performance and short/long-range FCS values in the WMHs group.** For IPS, we observed positive correlations in the left dorsolateral prefrontal cortex with long-range FCS, and in the right precuneus with short-range FCS. Warm colours represent positive correlations. FCS, functional connectivity strength; WMHs, white matter hyperintensities; IPS, information processing speed.

Based on the findings above, we further categorized the 18 FCS bins into two distinct groups: 0–100 mm (short-range FCS) and 100–180 mm (long-range FCS) (Fig. 2C). We generated the voxel-wise correlation maps to examine the associations between short-range and long-range FCS and cognitive performances in the WMHs group. For information processing speed, we observed positive correlations in the left dorsolateral prefrontal cortex with long-range FCS, and in the right PCUN with short-range FCS (Fig. 3). However, for the other three cognitive domains (i.e. episodic memory, visuospatial function and executive function), no significant correlations between the nodal FCS values (including short-range and long-range FCS) and neuropsychological *Z*-scores were observed.

## Discussion

In this present study, we systematically investigated the disrupted functional connectivity patterns in WMHs by using the R-fMRI data and graph-theory-based network analysis. Our findings revealed that WMHs selectively disrupted network hub regions, involving the dorsolateral prefrontal cortex, medial prefrontal cortex, PCUN, IPL, insula, thalamus and occipital lobe. Crucially, this disruption exhibited a dependence on connectivity distance. Furthermore, these disrupted network hub connectivities (particularly the long-range connections connected to the dorsolateral prefrontal cortex) positively correlated with the patients’ cognitive performance (i.e. information processing speed).

An increasingly recognized aspect of the human brain’s connectional architecture is that certain areas (known as hubs) serve as stations for information processing by connecting functional, distinct specialized systems.^20^ In this study, we noted that WMHs selectively disrupted network hub regions, mainly located in the dorsal attention network (DAN)/frontoparietal control network (FPCN) (e.g. dorsal anterior cingulate cortex, dorsolateral prefrontal cortex, posterior parietal cortex and insula) and default-mode network (DMN) (e.g. medial prefrontal cortex and precuneus). Although the pathophysiology of WMHs-related cognitive dysfunction is not fully understood, these findings support the disconnection of cognitive networks as a key mechanism that may result from vascular injury of the involved network hubs or from damage of interconnecting WM pathways.

Brain function maintenance depends on multiple networks that connect and interplay with each other to serve distinct functions.^32^ In cognitive neuroscience literature, the DAN, DMN and FPCN are widely recognized and well-established^33,34^ and correspond to the attention, executive function and information processing speed deficits most commonly affected by CSVD.^35^ The DAN, in conjunction with the FPCN, plays a key role in goal-directed attention under normal physiological circumstances.^36^ The DMN exhibits an anti-correlation with the DAN and is most active when the mind is at rest or engaged in self-referential, ‘stimulus-independent’ thinking.^37^ On the other hand, the FPCN is recognized for its flexible capacity to support both the DMN and DAN based on task demands, indicating a ‘regulatory’ role.^38,39^ The disruption of a single network may confer to the impairment of internetwork connectivity. Thus, based on these ‘cornerstones’ of functional connectivity under normal physiological circumstances, a disconnection hypothesis has emerged that postulates reduced connectivity within the DMN and FPCN, decoupling of neuronal activity along the anterior–posterior axis, and functional disconnection of the prefrontal cortex as neuronal correlates of cognitive decline in CSVD;^40^ and this hypothesis has been supported by many previous neuroimaging studies in CSVD.^12,41^ Intriguingly, to explore WMHs-related alterations in the functional hubs of whole-brain networks in this present study, we first employed voxel-based graph analysis and observed WMHs-related FCS decreases in several key nodes within the DMN and FPCN/DAN. This finding suggests a diminished role of these regions in global network function in patients with WMHs. Therefore, compatible with previous neuroimaging studies in CSVD, we further pointed out the notion that WMHs specifically disrupt brain hub regions within the DMN and FPCN/DAN, reflecting strategic ischaemic damage to cortico-cortical and cortico-subcortical circuitry. The dysfunction of these networks may provide clues to the mechanism of cognitive dysfunction in WMHs.

Diverse spatial patterns of short- and long-range functional connections have previously been documented.^20,22^ In this study, we further observed that WMHs specifically affected highly connected hub regions mainly involved the dorsal anterior cingulate cortex, dorsolateral prefrontal cortex, medial prefrontal, PCUN, IPL, insula and thalamus. Intriguingly, this impairment exhibited a dependence on connectivity distance, with the most pronounced disruptions observed in long-range connections. It is now increasingly recognized that long-range axonal fibres provide integration between distant regions and play a crucial role in supporting human cognitive function.^42,43^ Several postulated reasons may explain why long-range connections are especially susceptible to microangiopathic changes (e.g. WMHs). First, long-range neurons are crucial for maintaining network organization and efficiency, thus they are less numerous and consume more energy than short-range neurons.^26^ Further, compared to GM, WM (particularly the long-range fibres) is generally more susceptible to damage due to its lower blood flow^25^ and higher metabolic demands.^26^ Finally, it is well known that WMHs most commonly affect the periventricular and subcortical regions that contain long WM tracts connecting distant brain areas. Specifically, a recent explorative study using sophisticated fibre tracking methods has provided evidence that long-range fibres are more vulnerable to WMHs-related impairment compared to mid- and short-range fibres.^44^ Our study supports and expands on this finding. More importantly, we further found that the disruptions of hub connectivity (e.g. the long-range FCS in the left dorsolateral prefrontal cortex) positively correlated with the cognitive performance in WMHs patients, particularly the information processing speed. Therefore, we speculated that the WMHs may lead to a reduction especially in long-range connections, which may be a mechanistic mediator between WMHs and cognitive dysfunction (e.g. information processing speed).

Several potential limitations need to be further considered. In this present study, we sought to investigate how WMHs related to the connectivity patterns of brain hubs. Mixing participants might be problematic, as the severity of WMHs varies among individuals. We took this potential bias into account by recruiting the patients with WMHs Fazekas scores ≥ 3. Additionally, WMHs patients with microbleeds were further excluded from this study. However, we did not measure lacunar infarcts or perivascular spaces, which are other neuroimaging markers of CSVD. Further studies are warranted to quantify these markers together with WMHs to provide a more detailed picture of brain hub connectivity alterations associated with CSVD. Furthermore, we chose to measure WMHs using a published visual rating scale due to its easy and widespread use. However, the exact locations of white matter tracts were not considered, which might be valuable information for comprehending the neurophysiological correlates of cognitive dysfunction in CSVD. Finally, this study is cross-sectional in design and utilizes a relatively small sample size; however, future follow-up and large-sample studies are warranted to assess WMHs-related longitudinal alterations in the network hub connectivity and their long-term effects on cognitive dysfunction.

## Conclusion

Our findings emphasize that the disrupted hub connectivity patterns in WMHs are dependent on connection distance, especially longer-distance connections, which in turn predispose WMHs patients to worse cognitive function.

## Data Availability

The datasets generated for this study are available upon reasonable request from the corresponding author.
